# Application of mark-recapture to evaluate preventive road traffic injury policy

**DOI:** 10.5249/jivr.v6i2.479

**Published:** 2014-07

**Authors:** Davoud Khorasani-Zavareh, Maryam Bigdeli, Hosein Hatami, Ali Meshkini, Reza Mohammadi

**Affiliations:** ^*a*^Traffic Injury Prevention Research Center, Tabriz University of Medical Sciences, Tabriz, Iran.; ^*b*^Department of Clinical Science and Education, Södersjukhuset, Karolinska Institutet, Stockholm, Sweden.; ^*c*^Social Determinant of Health Research Center, Urmia University of Medical Sciences, Urmia, Iran.; ^*d*^School of Health, Shahid Beheshti University of Medical Sciences, Tehran, Iran.; ^*e*^Division of Social Medicine, Department of Public Health Sciences, Karolinska Institutet, Stockholm, Sweden.

In Iran, road traffics injuries (RTIs) rank as the second leading cause of death and are one of the most important public health problems,^1^ which affect health and life expectancy in the whole country. In order to deal with the problem, the Iranian Parliament has recently passed a national policy to reduce the number of fatal RTIs by 55% in all provinces within ten years.^2^ Any plan for preventive measures, however, should be based on sound RTI data.^3^ Different methods are available to estimate the number and rate of RTIs more accurately.^4^ These included cluster sampling and household surveys as well as relying on register systems. The first two are usually expensive and time-consuming and the last one usually suffers from under-reporting and consequently under-estimation of cases.^5^ To overcome this problem, the mark-recapture technique is a cost effective method, which relies on two or more incomplete and independent data sources and more importantly on the degree of overlap between them.^6^ The method will help to estimate the number and rate of real cases more accurately, in our case fatal RTIs. Accordingly, the aim of this study is to examine how the mark-recapture method can be applied to evaluate new government policy in terms of estimating fatal RTIs and following them up in Iranian provinces. 

In order to do that, two register systems called the Death Registry System (DRS) and the Forensic Medicine System (FMS) were used and data were extracted between one Iranian calendar and analyzed. The study base comprised persons who had died as a result of RTIs in West Azaribaijan Province (WAP). 

After combining two data sources, identical variables including name, age, sex and date of birth were considered to find common cases in the two data sources. After clean-up of the data set, the results indicated that DRS and FMS had registered 669 and 699 fatal RTIs cases respectively, all of which had occurred in the WAP region. Moreover, 447 common cases and a total of 921 aggregate deaths were found in the two sources. The mark-recapture method, however, estimated the number of fatal RTIs in the province to be 1046. This method showed that the coverage of DRS and FMS was 64% and 67%, and then, the correction factor was identified as 1.56 and 1.49 for the two register systems, respectively. Findings in this study are somewhat different from a previous study, which focused on cases where both death occurrence and residential address were in WAP.^5^ In addition, due to better coverage of death by FMS and regarding and more detail information about RTIs in the FMS, this system has potential to be surveillance, in order to help decision-makers for RTIs prevention. Application of mark-recapture indicated that there is around two-thirds completeness of real fatal RTIs in the DRS and FMS. However, despite under-reporting in FMS, the application of mark-recapture and employing a correction factor can be a cost-effective method of evaluation new injury prevention policy. 
